# *In-vitro* evaluation of apoptotic effect of OEO and thymol in 2D and 3D cell cultures and the study of their interaction mode with DNA

**DOI:** 10.1038/s41598-018-34055-w

**Published:** 2018-10-25

**Authors:** Tahereh Jamali, Gholamreza Kavoosi, Maliheh Safavi, Susan K. Ardestani

**Affiliations:** 1 Institute of Biochemistry and Biophysics, University of Tehran, Tehran, Iran; 2 Institute of Biotechnology, Shiraz University, Shiraz, Iran; 3 Department of Biotechnology, Iranian Research Organization for Science and Technology, Tehran, Iran

## Abstract

*Oliveria decumbens* is an Iranian endemic plant used extensively in traditional medicine. Recently, some studies have been performed on biological effects of Oliveria essential oil (OEO). However, to our knowledge, the anticancer activity of OEO has not been reported. Based on our GC/MS analysis, the basic ingredients of OEO are thymol, carvacrol, *p*-cymene and γ-terpinene. Therefore, we used OEO and its main component, thymol, to explore their effects on cell growth inhibition and anticancer activity. Despite having a limited effect on L929 normal cells, OEO/thymol induced cytotoxicity in MDA-MB231 breast cancer monolayers (2D) and to a lesser extent in MDA-MB231 spheroids (3D). Flow cytometry, caspase-3 activity assay in treated monolayers/spheroids and also fluorescence staining and DNA fragmentation in treated monolayers demonstrated apoptotic death mode. Indeed, OEO/thymol increased the Reactive Oxygen Species (ROS) level leading to mitochondrial membrane potential (MMP, ΔΨm) loss, caspase-3 activation and DNA damage caused S-phase cell cycle arrest. Furthermore, immunoblotting studies revealed the activation of intrinsic and maybe extrinsic apoptosis pathways by OEO/thymol. Additionally, *in-vitro* experiments, indicated that OEO/thymol interacts with DNA via minor grooves confirmed by docking method. Altogether, our reports underlined the potential of OEO to be considered as a new candidate for cancer therapy.

## Introduction

Breast cancer, a heterogeneous disease with diversity in morphological features and histological characteristics, is the most prominent leading cause of cancer death in women all over the world^1^. Despite the development of chemotherapy options for cancer, treatment has been limited because of numerous side effects that lead to failure of treatment^2^. Therefore, to overcome these deficiencies and minimizing the side effects, recognition of safe drugs especially with natural origin is essential^3–5^. Phytochemicals from medicinal plants have been considered as alternative approaches in cancer therapy and induction of the apoptotic death through various signaling pathways^4,6,7^. Many of these cytotoxic agents are effective via covalent or non-polar binding to DNA^8^. These agents inhibit cell survival in cancer cells via cell cycle arrest and induction of apoptosis.

Essential Oils (EOs), described as ‘the soul of plants’ are volatile complexes found in the aromatic plants and are used in pharmaceutical, and food industries for their anti-inflammatory, anti-microbial and anti-oxidant properties^9–11^. Additionally, anticancer activities of some EOs^12,13^ have been demonstrated in recent years. Terpenes and their oxygenated derivatives are the main components of EOs^14^. The EOs-mediated anticancer strategies recognized so far including apoptosis, cell cycle arrest, reactive oxygen and nitrogen species generation and DNA repair mechanisms. EOs reduce angiogenesis, metastasis and MDR (multidrug resistance) which make them potential candidates toward adjuvant anticancer agents. EOs affected tumor suppressor proteins, NF-*κ*B, Akt, Ap1, MAPK-pathway and detoxification enzymes activities such as superoxide dismutase, catalase, glutathione peroxidase, and glutathione reductase resulting in damage to the cells^15^.

*Oliveria decumbens*, a relatively less explored plant belongs to Apiaceae family, is an endemic plant growing in the south-western region of Iran. Oliveria essential oil (OEO) is used in Iranian traditional medicine for treatment of indigestion, diarrhea, abdominal pain and fever. Previous reports have confirmed the anti-microbial activity of OEO^14^, however, to our knowledge, there is no report about the anticancer activity of OEO. The basic components of OEO are thymol, carvacrol, *p*-cymene and γ-terpinene respectively. These components are useful plant materials with potent radical scavenging activity and display damaging or protective effects depending on the type of the cell and their concentration. There are some evidences on cytotoxicity effects induced by carvacrol on the human cancers such as cervical and lung cancer cell lines^16,17^.

In the present study, the effects of OEO and its main constituent, thymol, was studied on cancer cells survival and death signaling pathways in MDA-MB 231 cell lines as a model of breast cancer in 2D and 3D cultures. Additionally, the biological activity of the OEO/thymol was investigated *in-vitro*, regarding their interaction mode with dsDNA, as well as *in-silico*, by applying molecular docking simulations to consider the existence of possible interactions.

## Results

### GC/MS analysis

**Figure 1:**
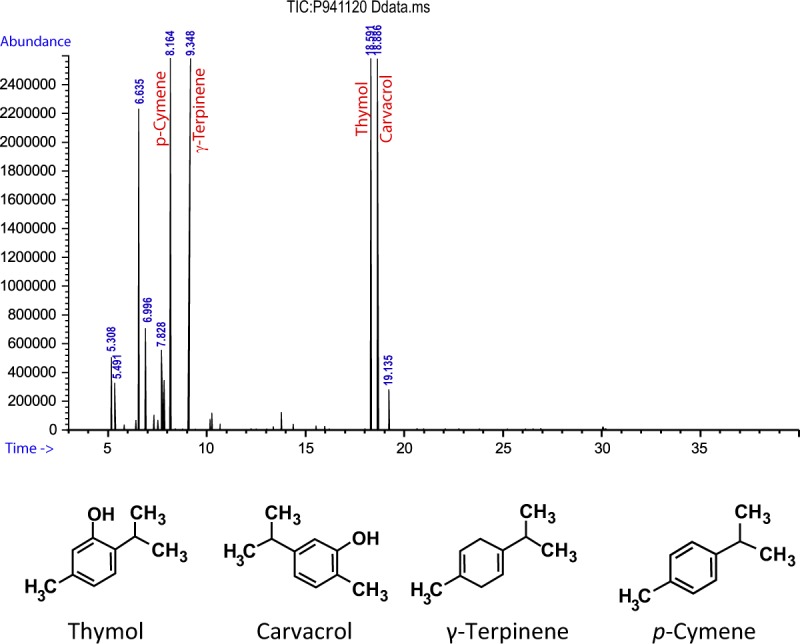
GC/MS chromatogram of OEO.

Qualitative phytochemical analysis of OEO by GC/MS revealed various components such as thymol (25.54%), carvacrol (23.12%), *p*-cymene (22.07%) and γ-terpinene (17.80%). The monoterpenoide phenols (carvacrol/thymol) constitutes the major components of OEO however OEO also is highly composed of monoterpenes (*p*-cymene/γ-terpinene) (Fig. 1, Table 1).

**Table 1:** Main chemical compositions of OEO.

Compounds	Retention index	Relative percent in OEO	Molecular weight	formula
Thymol	1289	25.54	150.217 g/mol	C10H14O
Carvacrol	1296	23.12	150.217 g/mol	C10H14O
*p*-Cymene	1026	22.07	134.21 g/mol	C10H14
γ-Terpinene	1059	17.80	136.24 g/mol	C10H16

Retention indices were determined using retention times of n-alkanes as standard on fused silica capillary HP-5 column that were injected after essential oil under the same chromatographic conditions.

### Spheroids generation

**Figure 2:**
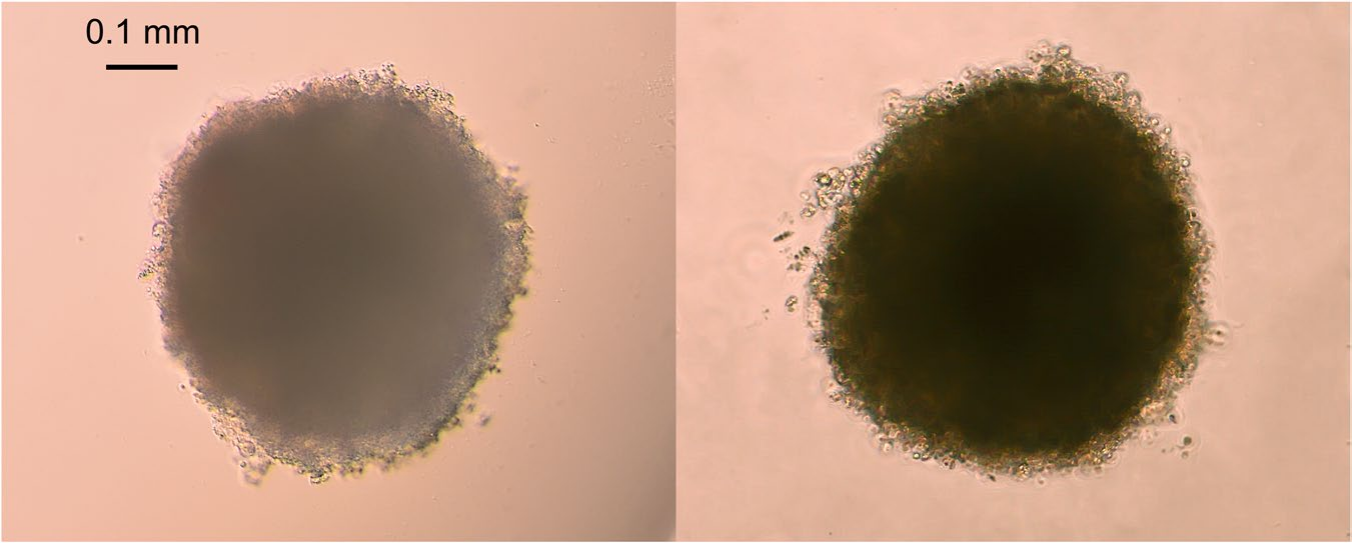
Spheroid formation in MDA-MB-231 (left) and MCF7 (right). they were imaged by inverted microscope.

The ability of 3D systems to resemble tumor-like microenvironment suggests the potential of 3D culture to provide more accurate cytotoxicity information for the drug discovery^18^. To study the effect of OEO/thymol on cellular spheroids, with combination of two methods, hanging drop and liquid overlay, proper spheroids were prepared. This is a convenient and quick method to form uniform spheroids. Indeed, spheroids created from 4000 MDA-MB-231 cells and 5000 MCF-7 cells gradually increased in diameter in 7 days. With consideration and analysis of phase-contrast images of spheroids, the average diameter of spheroids were calculated 500 ± 4 μm (Fig. 2).

### Induction of cytotoxicity by OEO/thymol in cancer cells monolayer (2D) and cell spheroids (3D)

**Figure 3:**
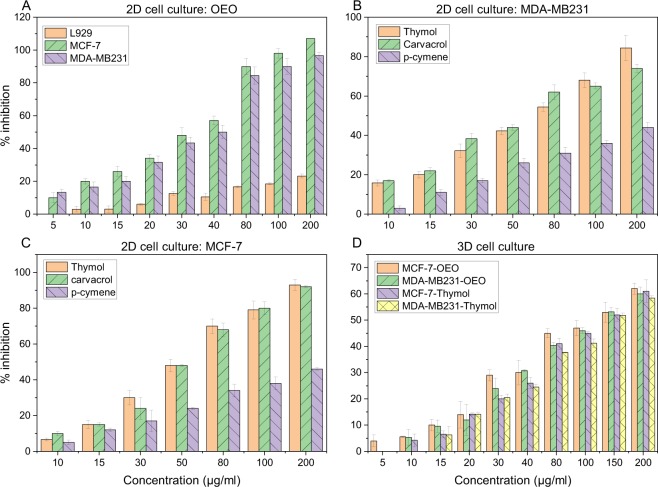
Growth inhibition of (**A**) L929, MCF-7 and MDA-MB-231 monolayers after treatment with increasing concentrations of OEO for 24 h (**B**) MDA-MB-231 monolayers after treatment with increasing concentrations of thymol, carvacrol and *p*-cymene for 24 h and (**C**) MCF-7 monolayers after treatment with increasing concentrations of thymol, carvacrol and p-cymene for 24 h D) MDA-MB-231 and MCF-7 spheroids exposed to increasing concentrations of OEO and thymol for 24 h. Cell inhibition percentage and IC_50_ was determined using the MTT assay. The results are the means ± SDs from triplate experiments (P < 0.05).

MTT assay is performed to detect cell viability and proliferation. It is based on the reduction of yellow tetrazolium MTT to a purple formazan by mitochondrial succinate dehydrogenase. Studying the growth-inhibitory effect of OEO on MDA-MB-231, MCF-7 and L929 cells in 2D monolayer culture by MTT assay showed the effect of OEO in a dose-dependent manner with IC_50_ (the concentration of OEO or thymol at which 50% of cell proliferations are inhibited) of 31.2, 27 and >250 μg/ml respectively after 24 h (Fig. 3A). Indeed, while OEO does not induce cytotoxic effect on normal fibroblast cells, is potently toxic in cancer cells and exhibits a concentration-dependent decline in viability. In addition, the inhibitory effect of thymol on the proliferation of MDA-MB-231 and MCF-7 cell line monolayers was determined in a dose-dependent manner and IC_50_ was found 56 and 47 μg/ml (Fig. 3B,C). For spheroids measuring 500 ± 4 μm in diameter, treatment with OEO exhibited IC_50_ of 128.5 and 117.5 μg/ml in MDA-MB-231 and MCF-7 spheroids respectively. While thymol induced the cytotoxicity in these spheroids with IC_50_ of 149 and 134.5 μg/ml respectively (Fig. 3D). Thus the OEO/thymol can diffuse and induce its cytotoxic effect in the spheroids. However, 2D monolayer and 3D cultures exhibited differential sensitivities to treatment^19,20^. Indeed, MDA-MB-231 and MCF-7 cells were less sensitive to OEO/thymol in 3D conditions compared to 2D monolayer cells. Therefore, the cell viability studies demonstrated that OEO/thymol is potent anti-proliferative compound in proliferating breast cancer cells, in a dose-dependent manner.

Additionally, in this study, the cytotoxic effects of two other components of OEO, carvacrol and *p*-cymene, on mentioned cell lines were studied. Our data showed that 24 h treatment with carvacrol decreases the viability of MDA-MB231 and MCF-7 monolayers in a concentration-dependent manner, with IC_50_ of 53 and 46.5 μg/ml respectively while the IC_50_ of *p*-cymene was approximately estimated to be 295.2 and 261 μg/ml in MDA-MB231 and MCF-7 cells (Fig. 3B,C). According to these results, carvacrol has the same cytotoxic effect, or even better, than thymol on the breast cancer cells while *p*-cymene inhibits the growth of these cancer cells in higher IC_50_ and therefore *p*-cymene has no significant effects on these cells.

### Apoptosis induction by OEO/thymol

#### Microscopic analysis

**Figure 4:**
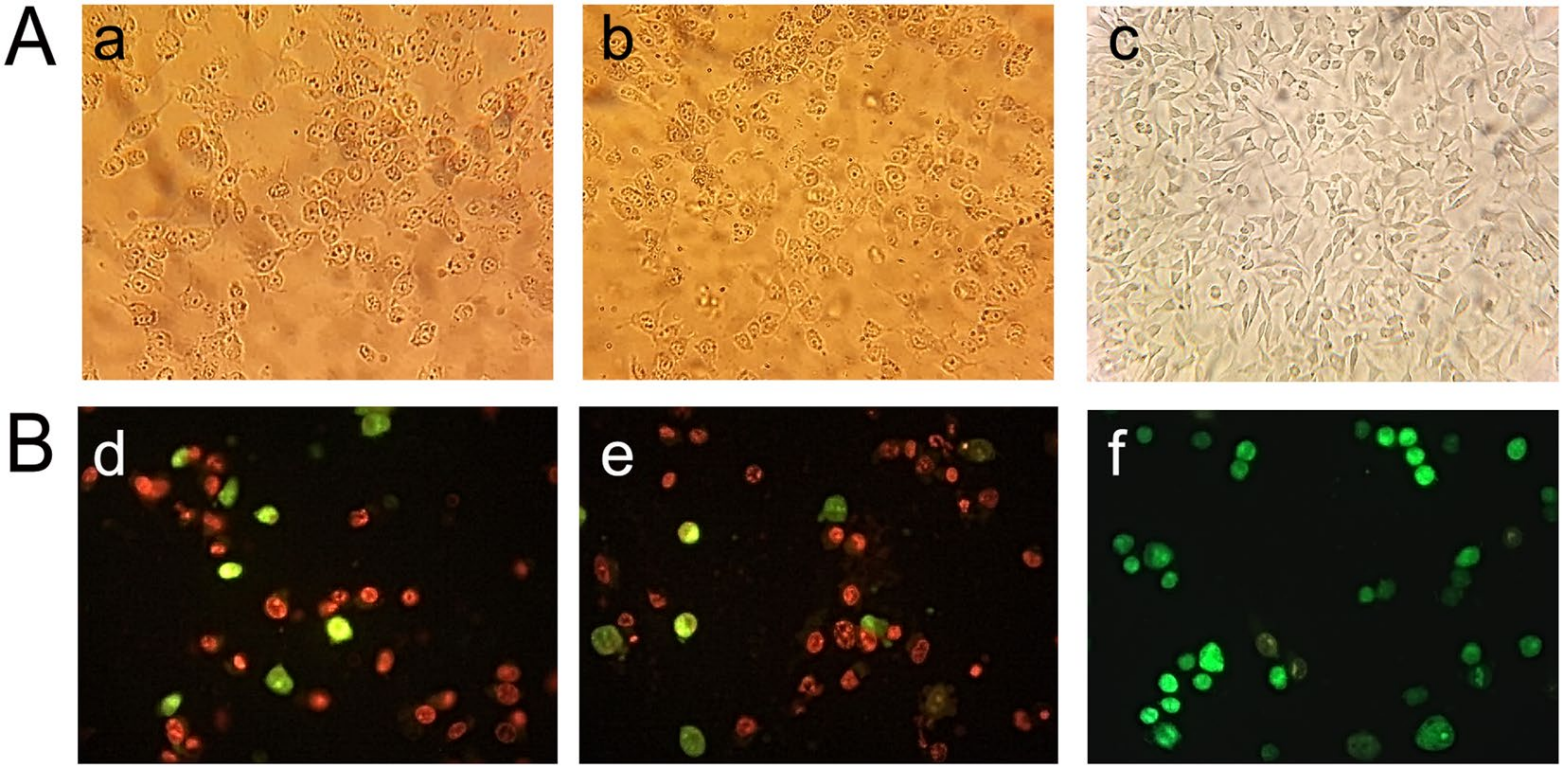
Microscopic studies: (**A**) Morphological analysis of MDA-MB231 under inverted microscope. (a,b) the cells exposed to IC_50_ of OEO and thymol for 24 h; with morphological alterations including loss of adhesion, rounding, and sporadic distribution (c) control cells; with typical shape and a growth pattern of patchy monolayer. (**B**) Fluorescence staining of MDA-MB231 cells (d,e) the cells exposed to IC_50_ of OEO and thymol for 24 h (f) control cells. The cells were stained using AO and EtBr and visualized with fluorescence microscopy.

Distinct morphological changes in cancer cells in the presence of IC_50_ of OEO/thymol for 24 h were observed by inverted microscope (Fig. 4A). Obtained images showed that treatment with OEO/thymol, results in cell rounding, detachment and floating, shrinkage and cytoplasmic vacuolation which characterize apoptosis in the cells.

To verify the apoptosis induced by OEO/thymol, fluorescence microscopy analysis of acridine orange (AO)/ethidium bromide (EtBr) stained cells was undertaken. This method with staining the cells, clearly showed apoptotic morphological changes in treated cancer cells. Green cells, stained only with AO, are viable cells, while late apoptotic cells, stained with EtBr, are orange and, finally green and orange cells with condensed chromatin, stained with both AO and EtBr, represent early apoptotic cells. In MDA-MB-231 cells treated with OEO/thymol, it was mainly shown that the mode of cell death is apoptosis characterized by staining due to chromatin condensation and loss of membrane integrity (Fig. 4B).

#### Annexin V/ propidium iodide (PI) staining

**Figure 5:**
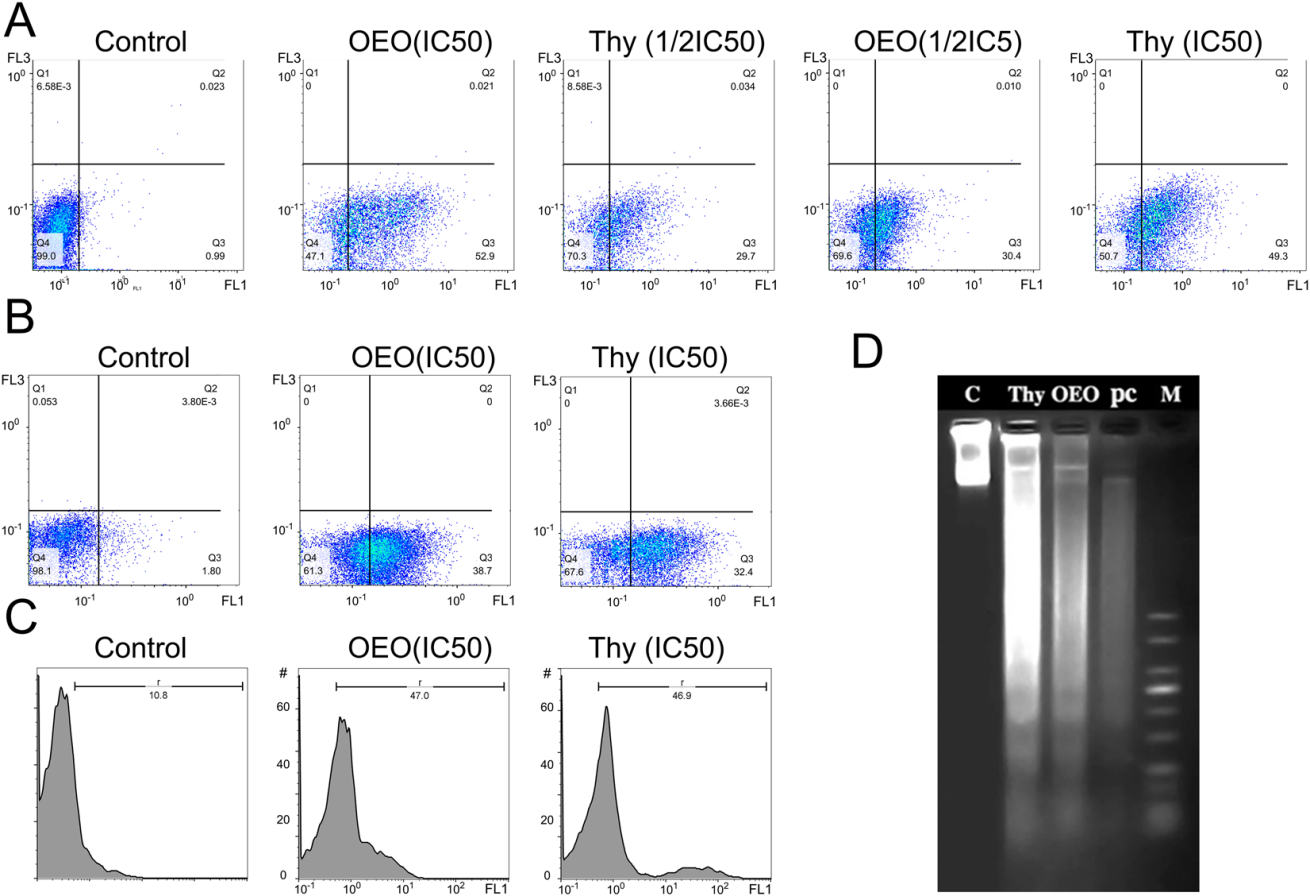
Annexin-V/PI analysis by flow cytometry in (**A**) MDA-MB231 monolayer cells (2D): control and treated cells with OEO (IC_50_ and 1/2IC_50_), thymol (IC_50_ and 1/2IC_50_). (**B**) MDA-MB231 cells separated from spheroids (3D): control and treated cells with OEO (IC_50_) and thymol (IC_50._). (**C**) DNA fragmentation in treated MDA-MB-231: TUNEL assay, apoptotic intensity of MDA-MB231 cells was determined by flow cytometry after TUNEL assay. (**A**) Shift of the population to the right in treated cells compared to control cells indicates the apoptotic cell population. (**D**) DNA fragmentation in treated MDA-MB-231: DNA laddering, Lane 1: Control, Lane 2: the cells treated with thymol (IC_50_); Lane 3: the cells treated with OEO (IC_50_). Lane 4: the cells treated with positive control (IC_50_) and Lane 5: molecular marker.

In healthy cells, phosphatidylserine (PS), is restricted to the inner leaflet of the plasma membrane and is exposed to the cell cytoplasm. Through apoptosis the membrane is ruptured and PS becomes exposed on the outer leaflet of the membrane. Annexin V, a 36-kDa calcium-binding protein, binds to PS and thus fluorescently labeled Annexin V (Annexin V-FITC) is able to detect PS in apoptotic cells. However, Annexin V can also access to necrotic cell’s membrane because these membranes have lost integrity and permit Annexin V to contact to destroyed plasma membrane. PI stains necrotic cells or cells in the late stage of apoptosis. Therefore, viable, early/late apoptotic and necrotic cells can be distinguished via co-staining of AnnexinV-FITC and PI. Hence, Annexin V binding and PI uptake considered by flow cytometry can quantify apoptosis in treated cells and identify the early apoptotic (Annexin V+/PI−) and late apoptotic/necrotic (Annexin V+/PI+) cells. The results of Annexin V/PI staining indicated that OEO/thymol in a concentration dependent manner induces early apoptosis (FITC-Annexin-V+/PI−) in MDA-MB-231 cells significantly after 4 h (Fig. 5A). In addition, with adding the IC_50_ of OEO/thymol on MDA-MB-231 spheroids after 24 h, apoptosis in spheroids were considered. The percentage of apoptotic cells measured by flow cytometry, confirmed the early apoptotic effects of components on spheroids (Fig. 5B). Therefore, it has been shown that OEO/thymol induces significant cell apoptosis in cancer cells.

### Induction of DNA fragmentation by OEO/thymol

#### DNA ladder

DNA fragmentation is a key event of apoptosis which distinguish apoptotic cells from necrotic ones. Nucleases like CAD (Caspases-activated DNase) play an important role in DNA cleavage resulting into a distinguishing ladder pattern on the agarose gel. In this study, obtained results showed that OEO, thymol and Doxorubicin (an inducer of DNA fragmentation) after 24 h significantly increase internucleosomal DNA cleavage in treated MDA-MB-231 cells which qualitatively is observed by DNA ladder formation on agarose gel (Fig. 5D).

#### TUNEL (Terminal Deoxynucleotidyl Transferase dUTP Nick End Labeling) assay

To confirm the apoptosis, double stranded DNA fragmentation was quantitatively evaluated by TUNEL assay using flow cytometry. By this assay which is a classical method for detection of DNA breakage by labeling the terminal end of nucleic acids, the effect of treatment of MDA-MB-231 cells with IC_50_ of OEO/thymol after 24 h on DNA fragmentation was investigated. Based on results, DNA fragmentation by OEO/thymol was estimated in treated cells about 4.3 fold of untreated control (Fig. 5C).

### Induction of apoptosis by OEO/thymol via a caspase-3 dependent pathway in cancer cells monolayer (2D) and cell spheroids (3D) culture

**Figure 6:**
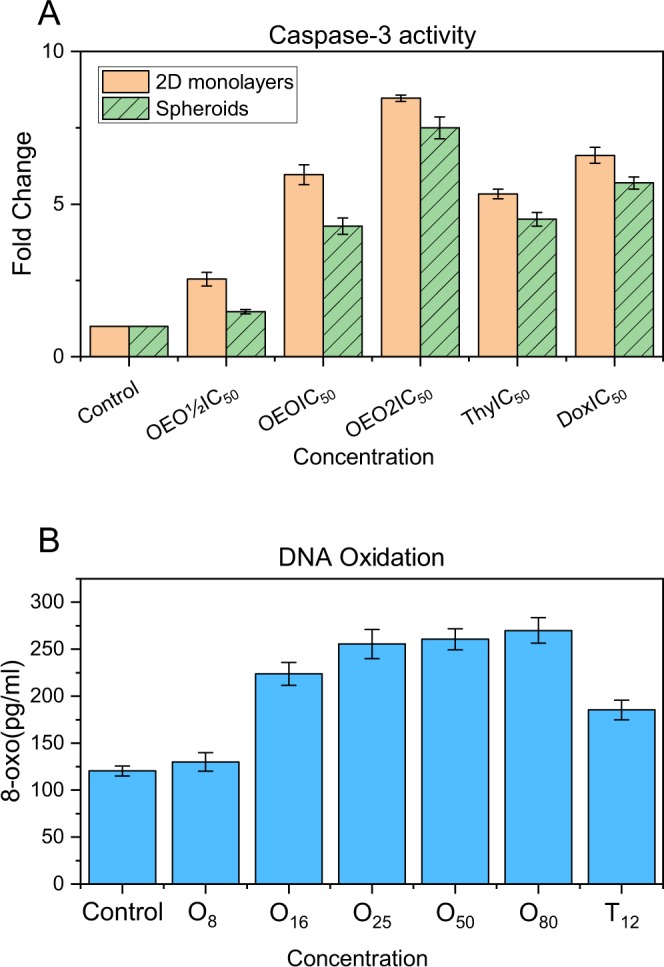
(**A**) Caspase 3 activity assay in control and treated cells with OEO (1/2IC_50_, IC_50_ and 2IC_50_), thymol (IC_50_) and Doxorubicin (IC_50_) in A: MDA-MB231 monolayer culture (2D) and MDA-MB231 spheroids (3D). The data were expressed in fold change with respect to untreated control groups. (**B**) Levels of 8-oxo-dG in the control and treated MDA-MB231 cells with various concentrations of OEO and thymol.

Caspase-3, key effector caspase in apoptotic signaling pathway, by cleaving a broad spectrum of the cellular substrates results in apoptotic cell death. Comparison of the absorbance of the chromophore p-nitroaniline (p-NA) (obtained from cleavage of caspase substrate (Ac-DEVD-pNA)) from the treated samples with the untreated control allowed determination of the fold increase in caspase 3 activity. In this research, detection of caspase-3 activity in treated cells and spheroids and comparison with negative controls exhibited that caspase-3 activity was efficiently increased in treated cells with OEO and thymol (IC_50_) about 6 and 5.3 fold respectively. In addition, the treatment efficiently induced apoptosis in 3D culture as well showed by increased caspase activity, but to a less extent (Fig. 6A). These results indicated that OEO/thymol induces apoptosis via a caspase-3 dependent pathway in cancer cells in monolayer and spheroid cultures.

### Reduction of Δψ_m_ in MDA-MB231 cell lines treated with OEO/thymol

**Figure 7:**
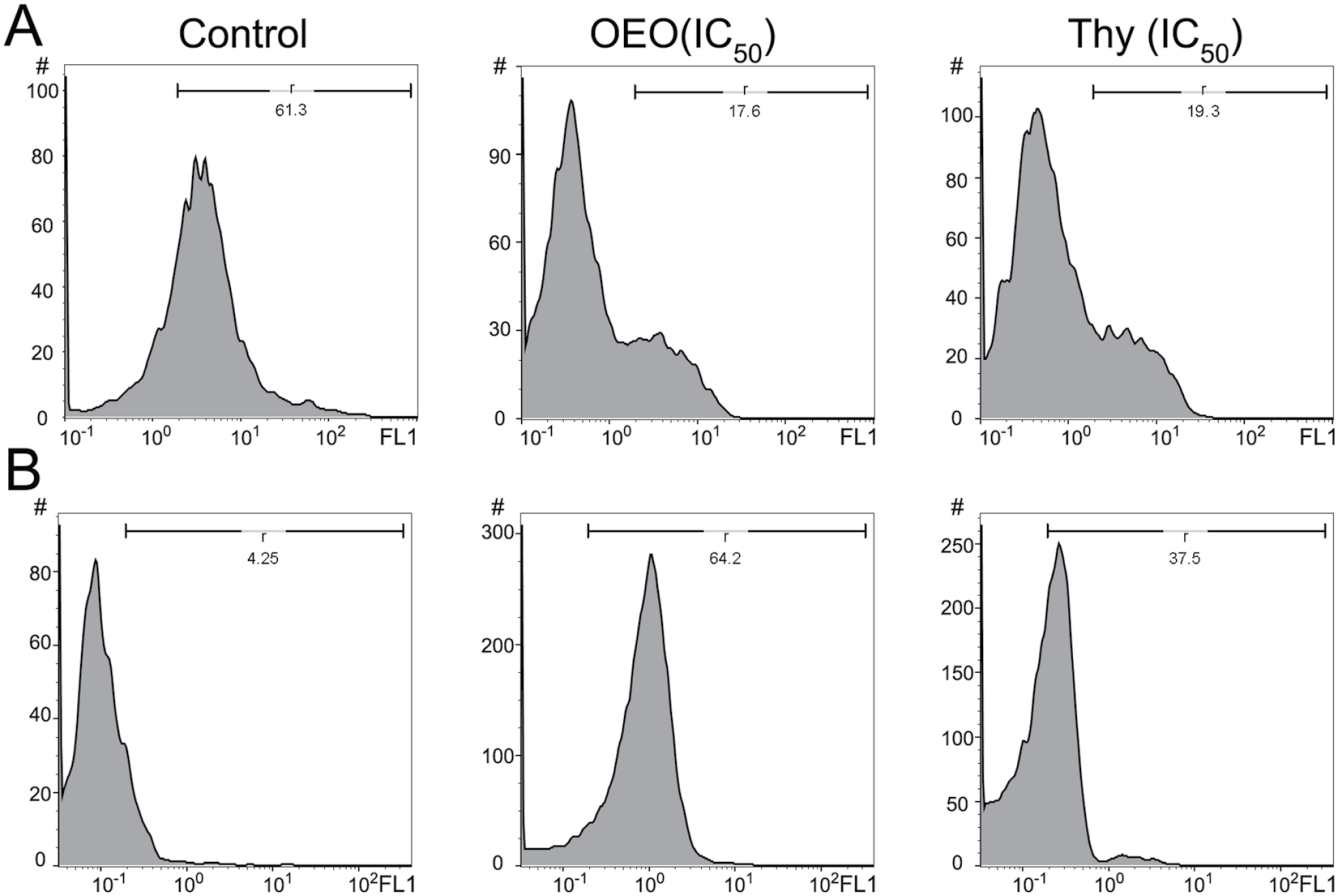
(**A**) Effect of OEO and thymol on Δψ_m_ in MDA-MB-231 control cells and treated cells with IC_50_ of OEO and thymol. The histograms reveal a left shift of peak demonstrated the decrease of Rh-123 fluorescence intensity because of the loss of MMP. (**B**) Effect of OEO and thymol on the formation of reactive oxygen species (ROS) in MDA-MB-231 control cells and treated cells with IC_50_ of OEO and thymol respectively. The histograms with a right shift reveal the increase of ROS level in treated MDA-MB231 cells with OEO and thymol.

ΔΨm is a sensitive indicator of the mitochondrial permeability; therefore to further assess whether OEO/thymol is involved in the mitochondrial-dependent apoptosis, MMP was evaluated in treated cells by Rhodamine 123 (Rh123) staining using the flow cytometer. As shown in Fig. 7A, the right peak, related to healthy cells, decreased in treated cells with IC_50_ of OEO and thymol rather than untreated cells while the left peak, related to unhealthy cells with the depolarized ΔΨm, significantly increased (increase in Rh123 fluorescence means depolarization). Quenching of Rhodamine is proportional to reduction of ΔΨm which is along with the increase in mitochondrial membrane permeability. These results showed that the mitochondrial pathway can be responsible for OEO/thymol-induced apoptosis.

### Induction of apoptosis via intracellular ROS generation by OEO/thymol

Accumulation of ROS plays a vital role in toxicity induced by antitumor compounds and thus regulation of cellular apoptosis^21^. Following the reduction of ΔΨm, which mediates intrinsic apoptosis pathways, and to determine whether ROS generation is responsible in anti-proliferative effects of OEO/thymol, the treated MDA-MB231 cells were monitored by staining 2′,7′-dichlorofluorescein diacetate (DCFH-DA) subjected to flow cytometry. Our results showed that OEO and thymol significantly induce the oxidative stress and elevate the levels of ROS about 15 and 9 fold respectively in treated MDA-MB231 cells (Fig. 7B). Therefore, our studies suggested that cell death induced by OEO/thymol can be related to ROS production.

### Determination of 8-oxo-dG (8-oxodeoxyguanosine) in MDA-MB-231 cells treated with OEO/thymol

Induction of ROS overload prompts oxidative DNA damage. Therefore, to study if the high ROS levels in treated MDA-MB231 cells caused oxidative DNA damage, we measured total cellular 8-oxoG, which is a major oxidized base lesion in genomic DNA. The results indicated that treated cells with OEO/thymol for 4 h are markedly contained in higher cellular 8-oxoG levels than untreated cells (131.6 pg/ml). In deed, 8-oxoG levels increased in treated cells in a dose dependent manner (Fig. 6B). Therefore, generally it was suggested that the OEO/thymol causes apoptosis through induction of oxidative DNA damage.

### Distribution of cell cycle in MDA-MB231 treated with OEO/thymol

**Figure 8:**
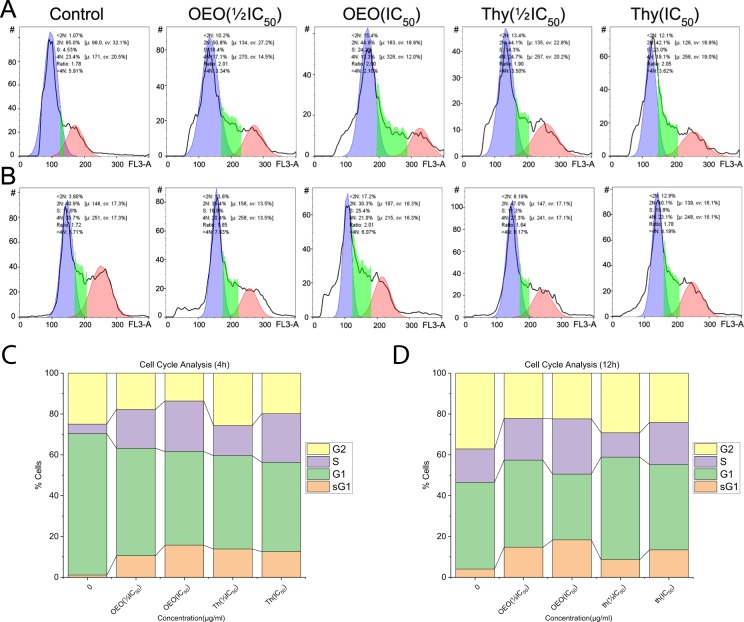
(**A**,**B**) Effect of OEO and thymol on DNA content of MDA-MB-231 cells after 4 h (top) and 12 h (bottom). (**C**,**D**) Measurement of cell cycle populations after treatment with OEO and thymol after 4 h and 12 h. Cell cycle analysis are done using a DNA intercalating dye, such as PI, to quantify the amount of DNA present in each cell.

Since the induction of apoptosis is correlated to the dysregulation of cell cycle, the effect of OEO/thymol on cell cycle in treated cells was investigated. Our results showed that the repartition of population changes in treated cells compared to untreated cells. Indeed, the proportion of cells in S-phase increased in cells treated with OEO/thymol after 4 h and 12 h, suggesting that OEO/thymol induces S-phase cell cycle arrest in MDA-MB231 cells in a dose and time-dependent manner. In addition, increase in the sub-G1 population confirmed the apoptotic effect of OEO/thymol in these cells (Fig. 8).

### Modulation of the expression of caspases and Bax/Bcl-2 ratio by OEO/thymol

**Figure 9:**
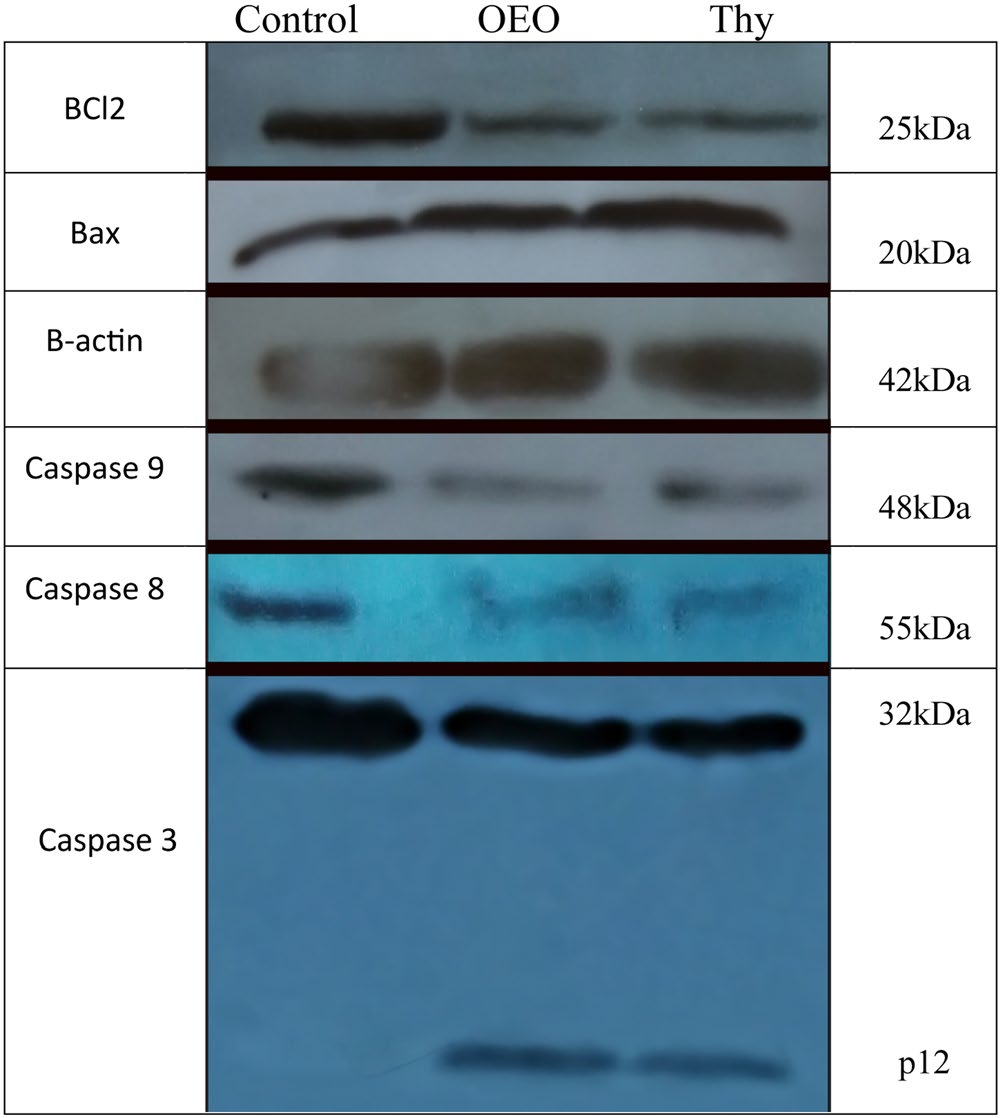
OEO/thymol-mediated apoptosis of MDA-MB231 cells involves a caspase-dependent mechanism and mitochondrial stress. MDA-MB231 cells were exposed to the IC_50_ of the OEO and thymol, and the expression of procaspases, Bax and Bcl2 were revealed by Western blot analysis.

As mentioned above, the apoptosis induced by OEO/thymol was accompanied by activation of caspase-3. To confirm if the antiproliferative effect induced by OEO/thymol is dependent on caspases, the expression level of procaspase-9, procaspase-8 and caspase-3 was investigated by western blot analysis. Furthermore, since an increase in the ratio of Bax/Bcl-2 (Bax: proapoptotic protein, Bcl2: antiapoptotic protein) is connected to ΔΨm reduction^22^, this relation was explored by investigation of Bax and Bcl-2 expression in OEO/thymol-treated cells. The expression level of procaspase-9, procaspase-3, procaspase 8 and Bcl2 was found to decrease in the treated cells (with IC_50_ of OEO/thymol for 24 h) compared with the control cells while the expression level of Bax and cleaved caspase-3 was observed to upregulate (Fig. 9). Therefore, OEO/thymol induces apoptosis through modulation of Bax/Bcl-2 ratio, decrease of procaspase-8, procaspase-9 and procaspase-3 levels and also the increase of cleaved caspase-3 level, and ultimately triggers intrinsic and maybe extrinsic apoptosis pathway with promoting the downstream signaling pathways to the death.

### OEO interacts with DNA via minor grooves

After DNA extraction from rat hepatocyte, the purity and integrity of DNA, were confirmed by nanodrop and gel electrophoresis.

#### UV–Visible Spectroscopy

**Figure 10:**
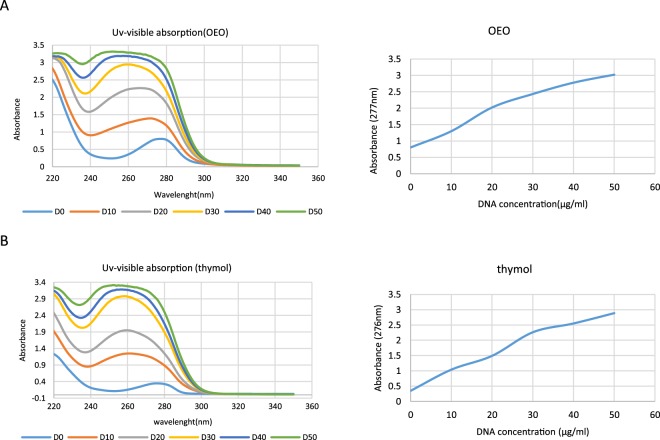
Interaction of (**A**) OEO and (**B**) thymol with dsDNA using UV–visible spectroscopy. UV–visible absorption spectra of OEO and thymol (50 µg/ml) in presence of increasing concentrations of dsDNA (0–50 µg/ml) in phosphate buffer (0.1 M with pH = 7.4).

UV–VIS absorption spectroscopy, has been used to investigate the interaction of DNA strands with small molecules by monitoring changes of UV–VIS absorption bands of DNA or small molecules. The UV spectra of OEO/thymol and the position of the peaks, with subsequent addition of DNA were considered to determine whether there is any interaction between DNA and the OEO/thymol. Indeed, amount of change or shift in the peak is linked with the strength of interaction. Outcome data showed that absorption peak decreased from 277 nm related to OEO to 253 nm by gradient addition of DNA to fixed amount of OEO (IC_50_) (Fig. 10A). And also absorption peak with adding the DNA to thymol shifted about 24 nm (Fig. 10B). The blue shift phenomena accompanied by hyperchromicity, approved the interaction of DNA to OEO/thymol via grooves and non-covalent interaction. However, since it was not observed any isosbestic point in the absorbance spectra and therefore there may be more than one type of binding, more experiments were required to confirm the binding mode^23^.

#### Fluorescence Spectroscopy

**Figure 11:**
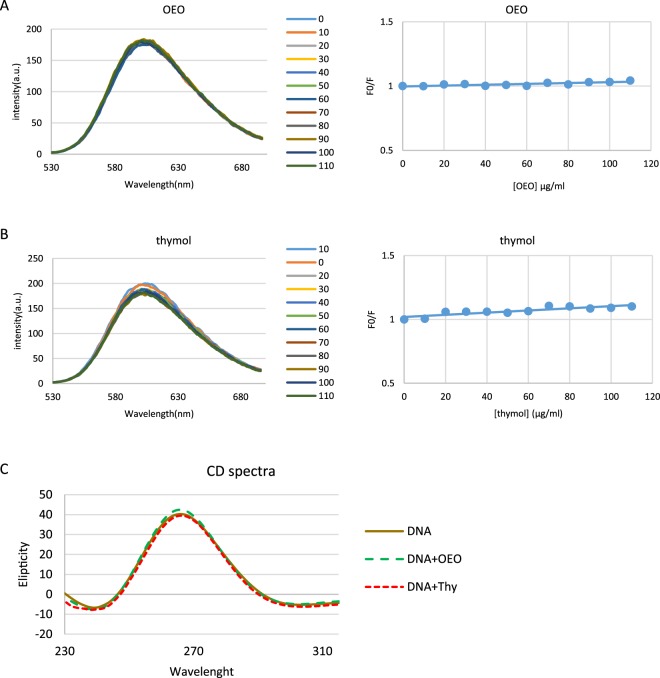
(**A** and **B**) Competitive displacement assays. Fluorescence titration of EtBr–dsDNA complex with increasing concentrations of (**A**) OEO and (**B**) thymol. No significant effect of OEO and thymol was seen on EtBr-dsDNA system. Right plots are Stern–Volmer plots for the mechanism of fluorescence quenching of EtBr–DNA by OEO and thymol. (**C**) Effect of OEO and thymol on CD spectra of dsDNA. CD spectra of dsDNA (50 µg/ml in phosphate buffer (0.1 M with pH = 7.4)) in presence of IC_50_ of OEO and thymol.

To more elucidate the interaction of DNA and OEO/thymol, fluorescence spectroscopy was performed. In this technique, to enhance DNA fluorescence at 600 nm initially fluorescent probe such as EtBr is bound to DNA by intercalation. In general, fluorescence quenching of EtBr–DNA complex is used to monitor the binding of molecules to DNA. Indeed, with adding the new ligand to the EtBr–DNA solution, substitution of EtBr with ligand occurs through a competitive reaction and finally results in fluorescence quenching. With adding increasing concentrations of OEO/thymol to EtBr–DNA solutions, the fluorescence emission spectra of the fixed amount of DNA and EtBr was estimated. The results showed that the fluorescence intensities of EtBr–DNA solutions does not affected significantly with the gradient adding of OEO/thymol suggesting these components bind to DNA in a non-intercalative mode (Fig. 11A,B). To rationalize the results, the ratio of fluorescence intensity before and after the addition of the quencher (F0/F) has been plotted (Fig. 11A,B) and in the following, to evaluate the fluorescence quenching efficiency, Stern–Volmer constant (Ksv) obtained from the slope of plot. According to the Stern–Volmer equation^24,25^:

$$\frac{{F}_{0}}{F}\,=\,1\,+\,{K}_{sv}\,[Q]$$

[Q] is the concentration of the quencher (OEO/thymol). As it is shown in Fig. 11, Stern–Volmer plot was linear which indicated that the process is either static or dynamic quenching. Meanwhile, K_SV_ was calculated 0. 50*10^2^ and 1.25*10^2^ M^−1^ for OEO and thymol respectively, which was not consistent with Ksv of intercalate mode. To more study the binding process, Kq, the bimolecular quenching rate constants, was calculated using the following equation:

$$Kq=\frac{{K}_{sv}}{\tau }$$

where *τ* is the average lifetime of DNA–EtBr in the absence of OEO/thymol and as to references is 10^−8^ s. Consequently, based on above equation, Kq was evaluated 0.5 × 10^10^ and 1.25 × 10^10^ M^−1^. Since these values for OEO/thymol are lower than the limiting diffusion rate constant (2 × 10^10^), the quenching process is dynamic rather than static.

#### Circular Dichroism (CD) spectroscopy

Circular dichroism spectroscopy is valuable to determine the mobility and orientation of intercalated ligands in dsDNA. The CD spectrum of dsDNA shows a positive peak at near 275 nm related to base stacking and a negative peak at near 245 nm related to the helical geometry of BDNA respectively^26,27^. On addition of OEO/thymol to a solution of DNA, slight changes in CD spectrum were detected. Indeed, because of the interaction between OEO and DNA, the intensity of both the negative and positive peak of DNA increased, while in interaction between thymol and DNA, intensity of the positive peak decreased and that of the negative peak increased (Fig. 11C). These results suggest that the presence of OEO/thymol slightly perturbs the stacking interaction and the right handed helicity of DNA. Since these changes are not significant, there may be a possibility that OEO/thymol binds to DNA through a groove mode.

#### Molecular modeling of ligands–DNA interaction

**Figure 12:**
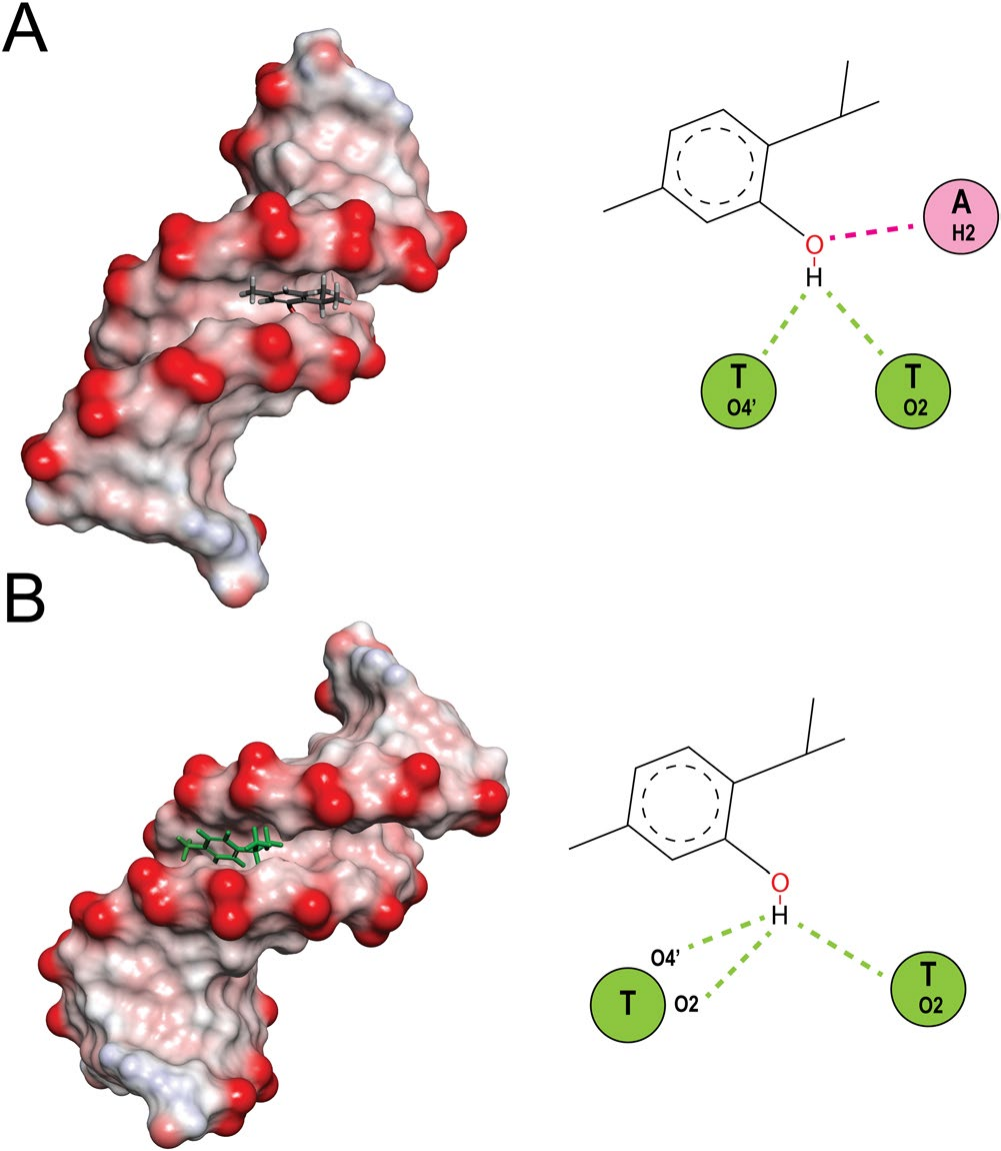
Molecular docked models of (**A**) thymol and (**B**) carvacrol with DNA (PDB 1D: 1BNA). Conventional hydrogen bonds and carbon hydrogen bonds have been labeled using green and pink dashed lines respectively.

As an important approach to predict the ligand/ receptor interactions, molecular docking was often used to offer the visual purpose for the binding mode of small ligands with DNA. The resulting binding energy of docked complexes was found to be −5.6 and −5.0 kcal M^−1^ for carvacrol, and thymol respectively. These results means carvacrol/thymol has the most frequent interaction with DNA. As shown in Fig. 12 carvacrol/thymol is entered into DNA minor grooves in Thymidine rich region. Two hydrogen bond (green dashed) formed between -OH group of thymol and O4’ associated with deoxyribose of T20 and also O2 of thymine nucleobase of T19 as long as 2.89 and 2.3 Å respectively. In addition -OH group of thymol formed a carbon-hydrogen bond (pink dashed) as long as 1.85 Å with H2 associated with adenine nucleobase of A18. While, in interactin of carvacrol with DNA, three hydrogen bond (green dashed) were revealed as long as 2.71, 2.55 and 2.15 Å between the O4’ associated with deoxyribose of T19 and O2 of thymine nucleobase of T19 and O2 associated with thymine nucleobase of T20 with -OH group of carvacrol respectively. The docking results suggested that carvacrol/thymol is prone to bind to the minor groove of DNA, and hydrogen bond forces may play an important role in the interaction between these ligands and DNA. However γ-terpinene and *p*-cymene also showed an interaction with the minor groove of DNA but with lower affinity. All these results confirmed the interaction of OEO main components with DNA.

## Discussion

Todays, many studies have been conducted to investigate the potential anticancer properties of diverse compounds to overcome cancer. Though, because of intolerance or resistance, many treatments have failed so far and progression of the disease is growing. Recently, interests in the use of medicinal plants have increased significantly due to their lesser side effects. The aim of this study was to initially evaluate the anticancer effects of OEO and thymol, a monoterpenoid phenol found in OEO (25.54%), followed by understanding the mechanisms underlying the activities of these components in cancer cell death. The GC/MS analysis of the OEO identified thymol, carvacrol, *p*-cymene and γ-terpinene as major components while other separated components including sesquiterpenes, phenylpropens, and non-phenolic portions were accounted for less than 15% of the oil. Although other studies have reported different amounts of OEO components than our analysis, in all the studies the maximum percentage belongs to thymol^28,29^.

Indeed, the differences in the percentage of the ingredients stem from differences in geographical variation, collection time, preparation process and other aspects. Several studies have indicated the anti-oxidant, anti-inflammatory, and anti-microbial activities of OEO and some its components such as thymol. All of OEO components possess a range of biological properties and effectiveness of OEO in the cell may therefore be caused by mechanisms related to several of these components. In fact, phytochemicals can have overlapping, inhibitory and complementary effects on the cell mechanisms such as cell proliferation, apoptosis or anti-oxidant activity. Therefore, the mixture of phytochemicals in OEO can provide different properties through a combination of their effects^30^. Thymol and carvacrol have also been found to possess some anti-cancer activity in 2D cancer cell culture, but its underlying mechanism has not yet been fully elucidated^17,31^. In this research, after the identification of main components of OEO obtained by hydrodistilation, thymol, as the main component with high percentage, was selected to be evaluate along with OEO itself. For the investigation of the growth-inhibitory effect of OEO/thymol, three cell lines, MDA-MB 231 (a triple negative breast cancer, invasive, and intermediate response to chemotherapy), MCF-7 (non-invasive human breast cancer cell line and responsive to chemotherapy) and L929 (mouse fibroblasts) were studied. Obtained results showed that OEO/thymol is capable to inhibit the proliferation of MDA-MB231 and MCF-7 cells in a dose-dependent manner. In addition, OEO did not display any significant toxicity in normal fibroblast cells (L929). Therefore, OEO possesses appropriate selectivity toward cancer cells. Furthermore, in this study, anti-proliferative effects of two other components of OEO, carvacrol and *p*-cymene, were investigated. The results demonstrated that carvacrol can obviously cause the growth inhibition of MDA-MB231 and MCF-7 cells with the similar ability of thymol or even better while *p*-cymene has no significant effect on cancer cells proliferation. Therefore, it is clear that better cytotoxic effects of OEO can be due to multi-component nature of OEO and a combination of additive and/or synergistic effects of several components such as thymol and carvacrol.

Despite of valuable information accumulated from conventional cell culture (2D), increasing failure rate of the designed drugs can be attributed to unreliable and defective results of 2D systems. Understanding the limitations of 2D cultures, recent studies have been conducted on the efficacy of drugs on cell spheroids as 3D models that provide a bridge between simple *in-vitro* monolayer cell cultures (2D) and the complex *in-vivo* real tumors^32,33^. Therefore, since 3D culture is able to recapitulate native tumor microenvironments^34–36^, MDA-MB231 and MCF-7 spheroids were generated and subsequently, treatment of the cell spheroids with OEO/thymol was performed. Our data from elicitation of the cell responses in 3D and comparison with conventional monolayer cultures showed the lower cytotoxicity of OEO/thymol in cell spheroids rather than on 2D culture as it was expected. Regardless of the specific action site of pharmacological agents, cell death induced by most useful drugs is mediated by triggering the apoptotic signaling pathways^37,38^. To study the inhibitory mode induced by OEO/thymol in the MDA-MB231 cells cultured in 2D and 3D systems, externalization of phosphatidylserine to the cell surface was investigated through incubation of cells with AnnexinV-(FITC) which confirmed apoptosis in treated cells, and it was compatible with the morphological changes of the treated cells under microscope^39,40^.

Looking for apoptosis induction properties of OEO/thymol, the DNA fragmentation was significantly observed in the treated cells. Activation of caspases, through selective cleavage of vital cellular substrates, results in apoptotic morphological changes and DNA fragmentation^41^. Accordingly, detection of caspase-3 activity in treated cells and spheroids showed that OEO/thymol leads to caspase-3 activation. Subsequent to the activation of caspase-3, cell triggers the enzymes such as DNase, resulting in DNA fragmentation and consequently the apoptosis. Cancer cells typically exhibit a high levels of ROS^42^. This may contribute to the progression of cancer, but may make cancer cells more vulnerable to extra ROS^43,44^. Recently, many drugs, mostly natural products, promote ROS overload specifically in cancer cells^45–47^. Enhancement of oxidative stress induced by current anticancer drugs is associated with blocking cancer development and induction of cell death^48^. However, the biochemical mechanisms linking ROS level to apoptosis is various and not exactly clear. ROS-induced oxidative stress directly or through DNA damage and cell cycle arrest, hyperpolarization of ΔΨm and cytochrome C discharge can provoke apoptosis^49^. In this research, we investigated whether OEO/thymol-induced apoptosis was mediated by ROS generation in MDA-MB-231 cells. Collectively, our results indicated that treatment of cells with OEO/thymol markedly increases ROS level in these cells. An important target of ROS is the guanine nucleotide pool as well as guanine nucleobase in DNA and RNA. The oxidative damage generates damaged guanine nucleotides such as 8-oxo- dGTP and 8-oxo-GTP. The existence of these molecules in genomic DNA is severely mutagenic and provokes cell death^50^. We showed that treatment of MDA-MB231 cells with OEO/thymol leads to accumulation of 8-oxo-dG. Although mitochondria membrane depolarization precedes the ROS production, ROS accumulation results in the loss of ΔΨm^51,52^. These procedures are associated with activation of mitochondrial apoptosis pathway. A common feature of early apoptosis is the active mitochondrial dysfunction, including changes in the redox potential of mitochondria^53,54^. Our findings demonstrated that OEO/thymol are able to decrease MMP of cells in a concentration-dependent manner and thus leads to mitochondrial apoptosis pathway. Induction of cell cycle arrest to prevent the cell proliferation is one of the effective ways used by cytotoxic drugs. These drugs play an anticancer role via arresting G0/G1, S or G2/M phases, thereby inhibiting cell growth, and eventually leading to apoptosis^55,56^. Following treatment of MDA-MB231 with various concentrations of OEO/thymol, results clearly verified that OEO/thymol has the potential to arrest MDA-MB231 cell lines at S-phase significantly. Therefore one of the toxicological mechanisms of OEO/thymol for inhibition of MDA-MB231 is the blockage of DNA replication, resulting in S-phase arrest and ultimately apoptosis. Although, some anticancer drugs initiate cell death through death receptors on the surface of the cell membrane (extrinsic pathway)^57^, most studies suggest a caspase-dependent pathway in mitochondria-based apoptosis (intrinsic pathway) by drugs^58,59^. However, the relationship between these paths has been observed at different levels. In addition, it is obvious that the simultaneous stimulation of the extrinsic and intrinsic pathways by drugs cause an increase in apoptosis. In the intrinsic pathway, the mitochondrial membrane permeability is a basic event resulting in caspase activation^60,61^. This permeability can be linked to ROS generation, DNA damage, and also proapoptotic members of the Bcl family. Bcl-2 family, including pro-apoptotic proteins (Bax, Bak, Bad, and Bcl-Xs) and antiapoptotic proteins (Bcl-2 and Bcl-XL) play an important role in regulating apoptosis induced by caspases. Indeed, upregulation of Bax and downregulation of Bcl-2 are associated with the enhanced levels of activated caspases and ultimately cell death^22^. Indeed, Increasing the level of the Bax leads to mitochondrial membrane damage, resulting in caspase-9 activation, an upstream initiator protease, followed by activation of caspase-3^62^. In extrinsic apoptosis pathway, stimulation of death receptors results in activation of the initiator caspase-8. This activated caspase-8 can amplify the apoptosis induced by cytotoxic drugs through cleavage of effector caspases such as caspase-3^63^. Moreover, caspase-3 feeds back on caspase-8, leading to its cleavage^64^. Caspase-3 is a frequently downstream effector caspase catalyzing the cleavage of numerous vital cellular proteins^65^. Based on this, the results of western blot analysis showed that in treated cells, the protein level of Bcl-2 decreases, while that of Bax increases, resulting in an elevation of the Bax/Bcl-2 ratio in MDA-MB231 cells. Furthermore, consideration of caspases clarified that the expression levels of caspase-9, 8 and 3 significantly decrease with treatment while the cleaved caspase-3 level increases. Collectively, these findings suggested that OEO/thymol not only stimulates the mitochondrial apoptosis pathway in MDA-MB231 cell lines, but also may amplify apoptosis through extrinsic pathway although because our components are nonpolar and able to cross the lipid membrane, we believe that rather than through the extrinsic pathway, these components by activating the intrinsic pathway and consequently caspase-3 cleavage, followed by activation of caspase-8 lead to more apoptosis.

The ability of a drug to interact with DNA is an important feature in the discovery of new anticancer agents^66,67^. Minor groove-binding and DNA intercalating drugs are interesting weapons in the battle against cancer. These agents target the DNA molecule and interfere with the cell cycle leading to cell death. Indeed, DNA interaction alters the cells fate by replication inhibition and/or transcription alteration. Minor groove-binding molecules usually bind non-covalently to DNA at A/T-rich regions and are capable to interfere with specific proteins in these regions. Furthermore, some of these agents inhibit the action of topoisomerases which are required mainly at the time of DNA replication and transcription. For example, anticancer drugs, such as distamycin A and pentamidine, bind to the DNA minor groove and form non-covalent complexes^68^. Since our results confirmed the arrest of cell cycle and apoptosis in treated cells by OEO/thymol, to elucidate whether OEO/thymol induces these properties based on interaction with DNA, several studies such as spectroscopic studies, CD spectral analysis and *in-silico* molecular docking were performed. Based on our results, by adding increasing amounts of DNA, alterations in UV-Vis spectra of OEO/thymol such as hyperchromicity were observed and confirmed non-covalent interactions in DNA-OEO/thymol complexes. In addition, competitive displacement assays with EtBr confirmed that OEO/thymol does not intercalate into the DNA base pairs and probably interacts with DNA through groove binding. These data were completed by CD spectral analysis, with observing the slight change at 275 nm positive band and 245 nm negative band. In *In-silico* molecular docking, the four main compounds of OEO, thymol, carvacrol, *p*-cymene and γ-terpinene were considered. Obtained data confirmed that thymol and carvacrol can bind to the minor groove of the DNA at thymidine rich region with suitable binding energy while *p*-cymene and γ-terpinene bind to the minor groove with lower binding affinity therefore in this interaction two components of thymol and carvacrol are important. Briefly, these findings showed that OEO/thymol is able to behave as an anticancer agent through interaction with DNA. Indeed, with creation of a complex between DNA and OEO/thymol, DNA replication is probably disrupted and cell cycle arrest occurs. In addition, binding of OEO/thymol to DNA may affect the transcription activities inside the cells and modify gene expression leading to altered regulation of cell proliferation and finally cell death.

Therefore, based on our findings, anticancer properties of OEO can be attributed to the presence of thymol in OEO. However, considering the inhibitory effect of carvacrol on cancer cell growth and also DNA interaction studies, the involvement of carvacrol in anticancer properties of OEO was confirmed. We should noticed that these findings are based on OEO components extracted in specific conditions. Another OEO is/is extracted in various conditions (such as the plant-growth region, the climate, the variety, harvest practices, post-harvest handling and extraction method) may not fully reflect these results and therefore may show some differences with our results. However, similar to our research, in the GC/MS analysis of OEO in previous studies, thymol, carvacrol, p-cymene and γ -terpinene have been known as the main components of OEO and, in all of these studies, thymol is in the highest percentage. This property increases the probability of similarity in the results and makes it easier to interpret the OEO results.

## Conclusion

**Figure 13:**
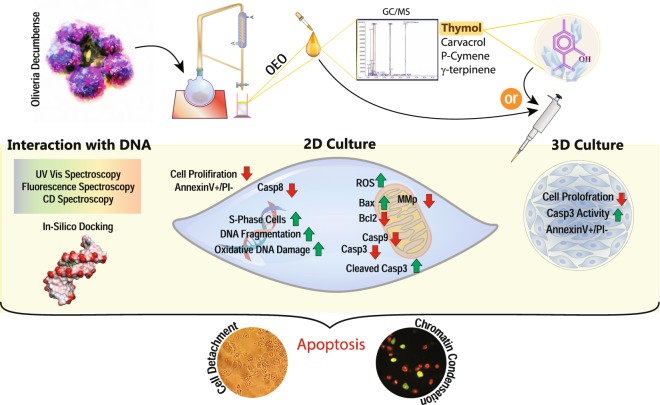
Schematic representation of OEO/thymol-induced apoptosis.

Our overall outcomes indicated that the OEO/thymol has significant anti-proliferative effects by inducing apoptosis in MDA-MB231 cells in 2D and 3D cultures, although 3D spheroids exhibited more resistance to the OEO/thymol. The present study provided a novel insight into the mechanism of action of OEO/thymol-induced apoptosis in cancer cells. Indeed, treatment indicated that OEO/thymol induces the cell death in MDA-MB231 cells through ROS mediated mitochondrial membrane depolarization, DNA fragmentation and caspase activity. We also showed that OEO/thymol can interact with DNA minor grooves and cause to cell cycle arrest and ultimately result in the intrinsic and less likely extrinsic apoptosis (Fig. 13). Collectively, despite some differences, based on the relatively similar results obtained from OEO and thymol, it can be inferred that some parts of the OEO effectiveness against the cancer cells is probably related to its high thymol content. However, lower IC_50_ value of OEO compared to thymol in inhibition of MDA-MB 231 cells proliferation, along with the anti-proliferative effect of carvacrol suggest that in addition to thymol, other components of OEO, such as carvacrol, are probably involved in OEO-induced anticancer effects. This conclusion was confirmed by observing the interaction of carvacrol with DNA minor groove *in-silico*. It worth noting the investigation of anticancer properties and mechanisms of carvacrol and other OEO components on MDA-MB231 cell lines could shed light on apoptotic events induced by OEO.

## Material and Methods

### Plant collection and OEO preparation

The aerial parts of *Oliveria decumbens* were collected from wild plants in the mountainous areas of Fars in Iran. OEO was extracted from the air-dried plants through hydrodistillation for 3 h using an all-glass Clevenger-type apparatus (Herbal Exir Co., Mashhad, Iran). The yield of essential oil was 2.5% (w/w). The essential oil was dehydrated over anhydrous sodium sulfate and stored at 4 °C until analyzed by GC/MS and was then used. The average of OEO density was obtained by digital balance (Acculab, Sartorius group, Germany) and reported about 1000 mg/ml^69^.

### Identification of the OEO chemical components

Essential oil was analyzed using an Agilent 7890 A series gas chromatograph (Agilent, Palo Alto, CA, USA) equipped with a flame ionization detector (FID) on a fused silica capillary HP-5 column (30 m × 0.32 mm i.d. and film thickness 0.25 µm). In this system, the split ratio of helium, as carrier gas, was 1/40 and injector volume was 0.1 μl. Temperature program was from 60 °C to 240 °C. Injector and detector temperature were set at 240 °C and 250 °C respectively. GC-MS was carried out using Agilent gas chromatograph coupled with Agilent 5975 C mass spectrometer equipped with a column HP-5MS. The temperature and carrier gas was the same as that of above and ionization source temperature was set on 280 °C. The identity of the oil constituents was assigned by comparison of Retention indices (relative to a homologous series of n-alkanes (C8-C25)) with those reported in the literature and the internal reference mass spectra library^13^.

### Preparation of OEO emulsion

1.0 ml of essential oil was added to 100 ml of water. Polysorbate-20 (100 µg/ml) was added to OEO-water mixture and the mixture was incubated at 35 °C for 24 h. At this time a milky emulsion was formed.

### Cell culture

Human mammary carcinoma cell line MDA-MB-231, MCF-7 and mouse normal fibroblast cell line L929 were purchased from National Cell Bank of Iran (Pasteur Institute, Iran). In both 2D and 3D culture, the cell lines were fed with RPMI-1640 (Gibco). This medium was supplemented with 10% FBS (Gibco) and 1% antibiotics (100 U/ml penicillin and 100 μg/ml streptomycin). Cells were maintained at 37 °C in a humidified incubator containing 5% CO2 and were passaged using trypsin/EDTA (Gibco) and phosphate-buffered saline (PBS) solution.

### Generation of multicellular spheroids

Hanging drop culture was performed by placing 50 μl drops containing approximately 4000 MDA-MB-231 cells and 5000 MCF-7 cells each on the inner surface of a 60 mm petri dish lid. Then the lid was gently inverted and placed on top of the plate containing 10 ml sterilized PBS to humidify the culture chamber. In this method, gravity force helps the cells to aggregate into a small spheroid at the bottom of drops^70^. After 3 days the formed spheroids were collected by wide-mouth tips. Then by liquid overlay method, formed spheroids were transferred and seeded into wells of poly-HEMA–precoated 96-well plates (which prepared using 0.5% poly-HEMA (sigma, 50 μl) dissolved in 95% ethanol and air dried for 3 days at 37 °C prior to use)^71^. Ultimately, spheroids with homogenous size were seeded into each well of the round bottom 96-well plate pre-coated with poly-HEMA filled by 200 µl complete medium. These plates were maintained in humidified incubator at 37 °C with 5% CO2. After 4 days, for measurement of spheroid size, we took some photos using invert microscope (Ax overt 25, Zeiss, Germany at 5 × and 10 × magnitudes) and then used ImageJ software to measure size and volume of spheroids. Our analysis showed spheroids with diameters ranging from 500–600 μm^13,72^.

### Cytotoxicity analysis in monolayer cells and spheroids

In monolayer culture, the single MDA-MB231, MCF-7 and L929 cell lines digested by trypsin/EDTA, were seeded at a density of 7 × 10^4^, 7.5 × 10^4^ and 5 × 10^4^ cells/ml respectively in 96-well plates filled with 200 μl medium per well for 24 h. For cytotoxicity studies, the cells were treated with various concentrations of OEO, thymol, carvacrol and *p*-cymene (0–200 μg/ml) for 24 h.

To investigate cell viability in spheroids, this test required some modifications. Following incubation time with OEO and thymol, MDA-MB231 and MCF-7 spheroids were transferred to a flat bottom 96-well plate, supernatant was removed and 200 μl MTT solution (5 mg/ml PBS; Sigma-Aldrich) was added to each well. Plates were incubated for 4 h at 37 °C. Subsequently, MTT were removed and 100 μl DMSO was replaced to ensure high solubility of formazan crystals. Then the absorbance was measured at 492 nm using an ELISA reader (Model wave xs2, BioTek, USA). Consequently, IC_50_ was calculated from the non-regression concentration-response curves^73^. In this study, polysorbate-20 was used as the solvent of OEO, thymol, carvacrol and *p*-cymene and also served as the negative control (final polysorbate-20 concentration in each well was about 0.5%).

### Fluorescent Staining

To evaluate the rates of cellular viability, fluorescent staining (with EtBr/AO (Sigma-Aldrich)) was performed. The permeability of EtBr and AO in the cells is based on the difference between membrane permeability into alive, apoptotic and necrotic cells. Monolayer MDA-MB231 were seeded in a 6-well cell culture plates containing 3 ml of complete medium. After 24 h, the cells were treated with IC_50_ of OEO and thymol. Following washing with PBS, a mixture of EtBr/AO (100 mg/ml) was added to the cells. The stained cells were visualized by a fluorescence microscope (Axioskop 2 plus, Ziess, Germany)^74,75^.

### Analysis of annexin V/PI-stained cells by flow cytometry

The apoptotic and necrotic treated MDA-MB-231 cells with OEO/thymol were identified using the Annexin V-FITC apoptosis kit (BioVision). Initially, the 3 × 10^6^ cells were seeded in a 6-well cell culture plate containing 3 ml of complete medium. After 24 h, the cells were treated with IC_50_ of OEO/thymol for 4 h. Then cells were harvested, washed and labeled with PI and FITC according to the manufacturer’s protocol. Finally, Annexin V-FITC/PI stained cells were analyzed using a flow cytometry. (Partec PAS, Germany)^76^. In this assay, we counted about 40000 and 20000 cells for each sample related to monolayer culture and spheroids respectively.

### DNA laddering assay

MDA-MB-231 cells were seeded in 60 mm dishes, and then treated for 24 h with OEO/thymol and Doxorubicin as positive control with IC_50_ doses. DNA extraction was performed according to standard phenol/chloroform extraction procedure. Extracted DNA was dissolved in TE buffer, and loaded on a 2% agarose gel. Finally the gel was stained with EtBr and photographed using a gel doc system for DNA visualization^77,78^.

### TUNEL Assay for *In Situ* apoptosis detection

Apoptotic DNA degradation was detected by the TUNEL assay *In Situ* Cell death Detection Kit (Roche), according to the manufacturer’s instructions. The cells (3 × 10^6^cells/well) were cultured in 6-well plates. After treatment with OEO/thymol for 24 h, cells were washed with PBS, fixed in 4% paraformaldehyde solution for 1 hour at room temperature in the dark. Following washing with PBS, the cells were incubated in permeabilization solution containing 1% Triton X-100 and 0.1% sodium citrate for 20 minutes on ice. Then DNA was labeled by incubating the cells with TUNEL reaction mixture (Tdt enzyme and fluorescein-conjugated dUTP) in a humidified air for 60 min at 37 °C in the dark. Finally the percentage of TUNEL-positive cells were determined from the histograms by flow cytometry^79,80^.

### ROS assay

The intracellular ROS was measured using DCFH-DA (Sigma-Aldrich). This molecule diffuses through cell membranes and is hydrolyzed by intracellular esterase to liberate the free acid (2′, 7′ dichlorodihydrofluorescin (DCFH2)) which is trapped in the cells. DCFH2, upon reaction with oxidizing species, forms the highly fluorescent compound 2′, 7′- dichlorofluorescein (DCF). Thus, the fluorescence intensity was proportional to the amount of hydrogen peroxide produced by the cells. MDA-MB-231 cells were exposed to OEO/thymol for 12 h. After washing with PBS, the cells were incubated in DCFH-DA (20 μM) and kept in the dark for 15 min, and the intracellular fluorescence was measured using flow cytometry (FACSCalibur, BD Biosciences) with excitation and emission settings of 485–495 nm/525–530 nm, respectively^81,82^.

### ΔΨm assay

Cells stained with Rh123, a cationic voltage-sensitive probe, were used for determining changes in mitochondrial transmembrane potential. Rh123 reversibly accumulates in mitochondria and was used to detect changes in ΔΨm. For the quantification of ΔΨm, MDA-MB231 cells were seeded in a 6-well cell culture plate (5 × 10^5^ cell/well) containing 3 ml of complete medium. After 24 h, the cells were treated with IC_50_ of OEO and thymol for 12 h. Then cells were harvested and pelleted by centrifugation, followed by resuspension with 1 mL of Rh123 (50 µM). Cell suspension was gently mixed and incubated for 20 minutes at 37 °C in the dark. The cells were then washed twice with PBS to remove extracellular Rh123. Finally, the fluorescent intensity of the Rh123 in stained cells was measured by flow cytometry using 488 nm laser excitation and a 525–530 nm emission filter^83,84^.

### Caspase-3 activity assay

Caspase-3 activity in the cells was measured using the caspase-3 colorimetric activity assay kit (BIOMOL International, USA). This assay is the based on spectrophotometric detection of the p-NA after the cleavage of the synthetic peptide Ac-DEVD-pNA, as caspase-3 substrate. About 3 × 10^6^ MDA-MB-231 cells were exposed to various concentrations of OEO, thymol and doxorubicin as positive control (50 µg/ml) for 4 h. Briefly, the cells were washed with cold PBS and lysed with the cell lysis buffer [PIPES, KCl, mgcl_2_, DTT, EDTA, EGTA containing PMSF, antipain, leupeptin, and aprotinin] on ice. The cell lysates were centrifuged (12000 g for 5 min at 4 °C), and the supernatants were collected. The protein concentration of supernatant was estimated by using the Bradford method. Finally, Equal amounts of protein (100 µg), 5 µL of colorimetric caspase-3 substrate (AcDEVD-pNA, 2 mmol/L) and assay buffer [Nacl, HEPES, DTT, CHAPS, EDTA, Glycerol] were added to each reaction mixture, and the reaction mixtures were incubated for 3 h at 37 °C and finally the absorbance was measured using a microplate reader at 405 nm^85^. Caspase-3 activity was expressed as the fold change in activity in treated cancer cells in comparison to the untreated controls.

### DNA oxidation analysis

8-Oxo detection Elisa kit (Cayman chemical 589320) is designed for measuring the concentration of the 8-Oxo-dG in DNA which is a frequently used biomarker of oxidative DNA damage and oxidative stress. For this assay, MDA-MB-231 cells was treated for 4 h with various concentrations of OEO and thymol. Genomic DNA of treated and untreated cells were extracted according to standard phenol/chloroform extraction. Approximately 5 μg of DNA from each sample was digested with nuclease P1 (sigma N8630) and incubated with alkaline phosphatase (NEB M0290S) and finally according to manufacturer’s protocol exposed to 8-oxo detection Elisa kit (Cayman chemical 589320). The amounts of 8-oxo were then assessed in pg/ml^86^.

### Cell cycle phase distribution Analysis of MDA-MB-231 Cells

Flow cytometry analysis was performed to define the cell cycle distribution. MDA-MB231 Cells were treated with various concentrations of OEO and thymol. After 4 h and 12 h, the cells were harvested, washed and fixed with 70% ethanol for 24 h. Cells were centrifuged and ethanol was removed. Afterward, Cells were stained for total DNA content with a solution containing 20 μg/ml PI and 50 μg/ml RNase I in PBS for 30 min at 37 °C in the dark. Finally, stained cells were analyzed by flow cytometry and the percentage of cells in the G0/G1, S and G2/M phases was measured using the FlowJo software Version 7.6.1^87,88^.

### Western blot analysis

Immunoblotting or western analysis of protein expression in cells is the reliable analytical method for assessing molecular biological roles. Treated and untreated cells were lysed in an ice-cold lysis buffer [PIPES, KCl, mgcl2, DTT, EDTA, EGTA containing PMSF, antipain, leupeptin, and aprotinin]. The lysates were hardly spinned for 10 mins and passed 10–15 times through an insulin-needle to facilitate cell breaking-up and liquefy the viscous lysate. All process was performed on ice. The cell lysates were centrifuged (12000 g for 5 min at 4 °C), and the supernatants (containing cytoplasmic proteins) were transferred to a new tube. Finally the concentration of proteins were quantified using the Bradford Assay. Equal amounts (20 μg) of protein lysates from each sample were separated by SDS–PAGE. The proteins were then transferred onto PVDF membranes. The membranes were blocked with 5% skim milk for 1 h and then incubated with primary antibodies for 12 h. The following primary antibodies were such as anti-caspase-9 (ab32539), anti-caspse-8 (ab119809), anti-caspase-3 (ab179517), anti-bax (ab32503), anti-bcl2 (ab32124) and anti-beta actin (ab8226). The membranes were then thoroughly rinsed and incubated with species-matched HRP-conjugated secondary antibody for 3 h. The unbound antibody was washed off. The bound antibodies were then detected by developing the film^89^.

### UV-Visible spectroscopy

UV–Visible absorption spectroscopy is one of the most commonly employed techniques for studying DNA interaction with small ligand molecules. Initially, Isolated DNA (according to Invitrogen protocol) from rat hepatocyte (dsDNA) was prepared in Na2HPO4-NaH2PO4 (0.1 M) buffer. Quantity and quality of DNA was evaluated by Nano Drop (thermoscientific-USA) and UV-Visible spectrophotometer (Cary 100 Bio-model, Australia). UV–visible absorption spectra of OEO/thymol (50 µg/ml) in presence of increasing concentrations of dsDNA (0–50 µg/ml) in phosphate buffer and also the absorption spectra for the free DNA (50 μg/ml), and in presence of increasing concentrations of OEO/thymol were recorded. The spectroscopic experiments were performed at 298 K^90–92^.

### Fluorescence quenching experiments

Dye competitive displacement assays were conducted using a DNA binding probes (such as EtBr). In EtBr displacement assay, constant concentrations of DNA (50 μg/ml) and EtBr (2.6 μM) was titrated with varying concentrations of OEO and thymol. The excitation and emission slit widths (each 10 nm) and scan rate were maintained constant for all the experiments. OEO/thymol emission spectra were recorded from 530 to 700 nm with a fixed excitation at 500 nm at 310 K using a Carry Eclipse fluorescence spectrophotometer^93^.

### CD spectral measurements

CD spectra of DNA extracted from rat hepatocyte alone (100 μg/ml) and in the presence of some concentrations of OEO/thymol were recorded with CD spectrophotometer (CD 215, Aviv, USA) equipped with a quartz cuvette with a path length of 1 cm. All spectra were studied in far-UV range^94,95^.

### Molecular modeling of OEO components with DNA

Since the main components of OEO are thymol, carvacrol, p-cymene and γ-terpinene, in continues of our study on DNA interaction, these ligands were chosen for molecular modeling with DNA. The 3D structure of thymol, carvacrol, p-cymene and γ-terpinene was downloaded from http://www.chemspider.com, and B-DNA structure was downloaded from the Protein Data Bank (PDB ID: 1bna)^96^. The molecular docking was accomplished by AutoDockTools-1.5.6/Vina from The Scripps Research Institute (http://autodock.scripps.edu/references)^97^. For docking, the receptor and ligands files were converted into PDBQT format, all water molecules were removed, and Gasteiger charges and polar hydrogen atoms were added to the DNA file using MGLTools (version 1.5.6). The grid box dimensions were selected to be sufficiently large to cover complete chains of DNA. ADVina shows the docking scores as binding free energy (Δ*G*). And ultimately all figures were visualized using PYMOL software^98–101^.

### Statistical Analysis

Experimental data processing was carried out using Microsoft Excel 2013 software and results were presented as mean+− standard deviation of three or more independent experiments. The significant differences between means were determined by t-test when statistical significance was P value ≤ 0.05.

## Electronic supplementary material

SI- Full length blots
