# Supplementary material for: In-vitro evaluation of apoptotic effect of OEO and thymol in 2D and 3D cell cultures and the study of their interaction mode with DNA
Source: Sci Rep. 2018 Oct 25;8:15787. doi: 10.1038/s41598-018-34055-w (PMC6202332; doi:10.1038/s41598-018-34055-w)
Supplement: Supplementary file 1 — SI- Full length blots [file 41598_2018_34055_MOESM1_ESM.pdf]

Supplementary Information

In-vitro evaluation of apoptotic effect of OEO and thymol in 2D and 3D cell cultures and the study of their interaction mode with DNA

Tahereh Jamali[1], Gholamreza Kavoosi[2], Maliheh Safavi[3], Susan K. Ardestani[1*]

1. Institute of Biochemistry and Biophysics, University of Tehran, Tehran, Iran
2. Institute of Biotechnology, Shiraz University, Shiraz, Iran
3. Department of Biotechnology, Iranian Research Organization for Science and Technology, Tehran, Iran
** Corresponding author: ardestany@ut.ac.ir*

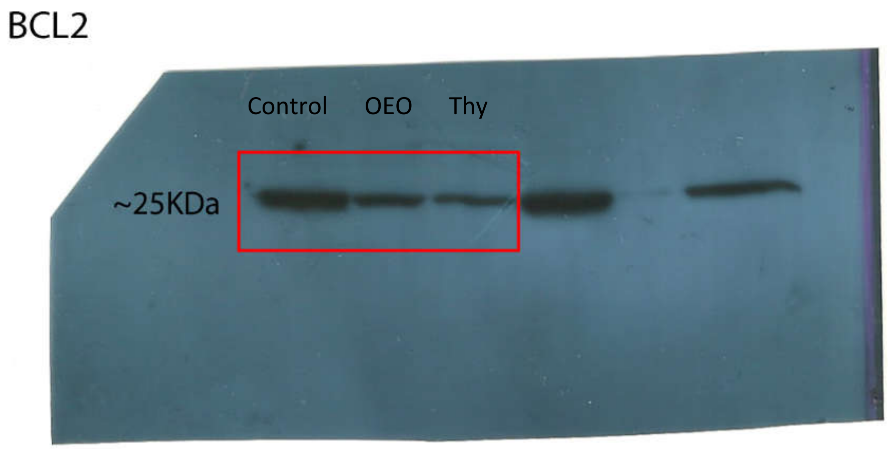

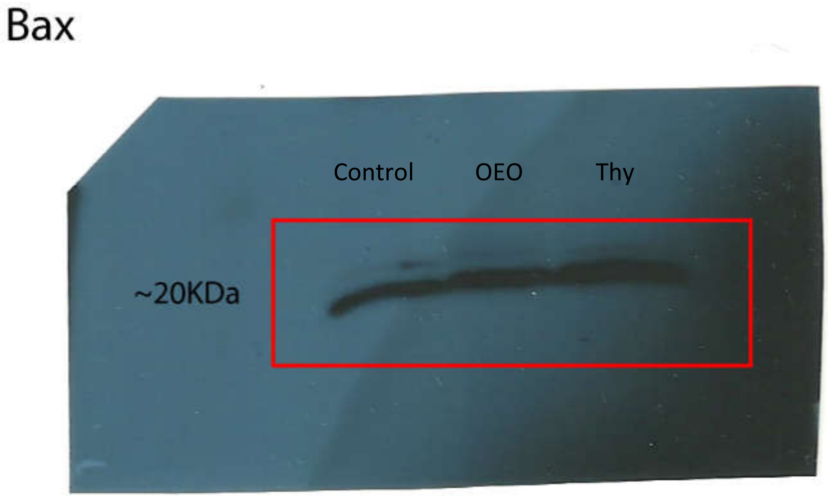

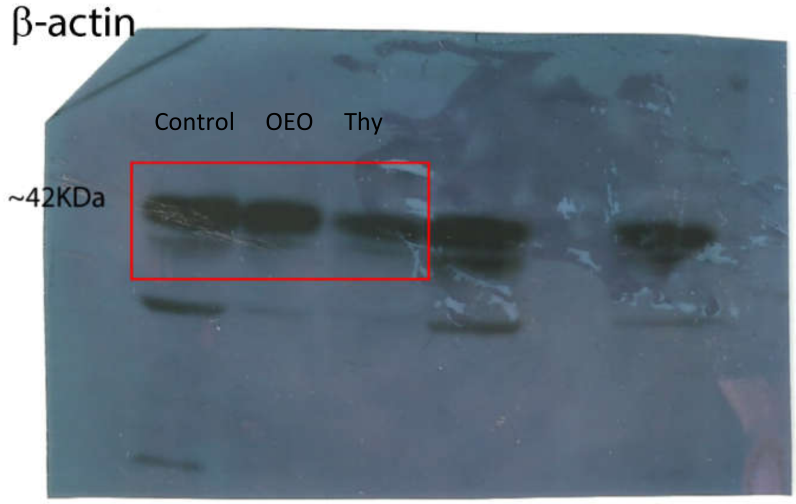
β-actin
Control
OEO
Thy
~42KDa

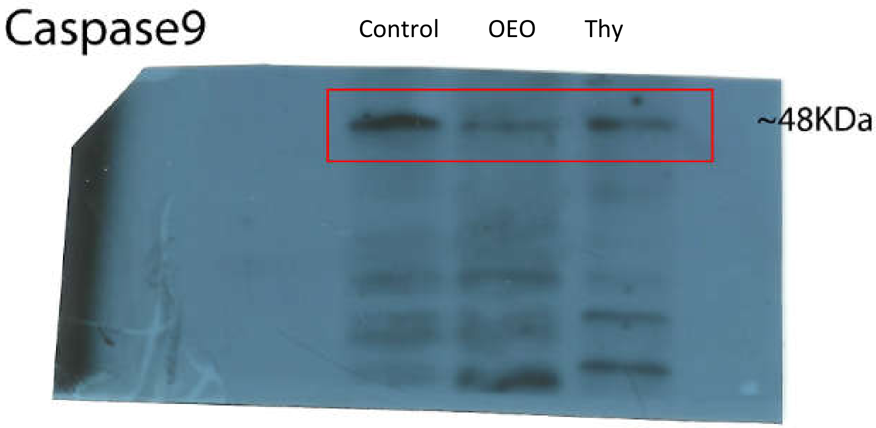
Caspase9
Control
OEO
Thy
~48KDa

Caspase8

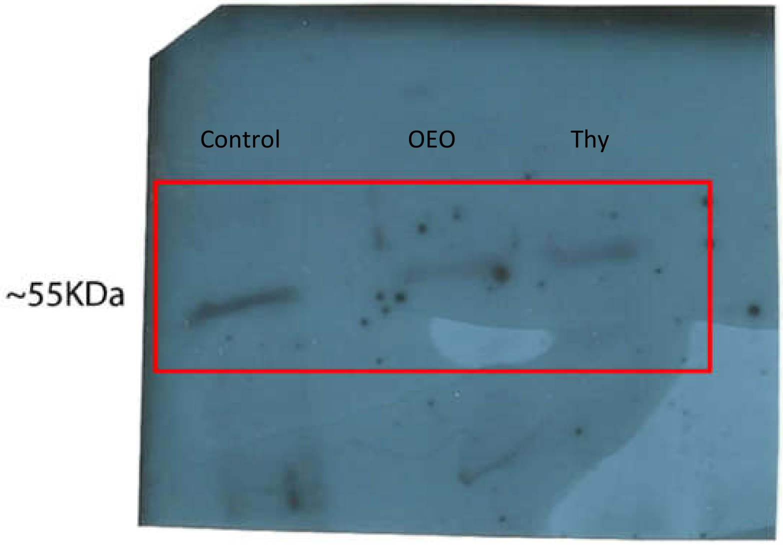

Caspase3

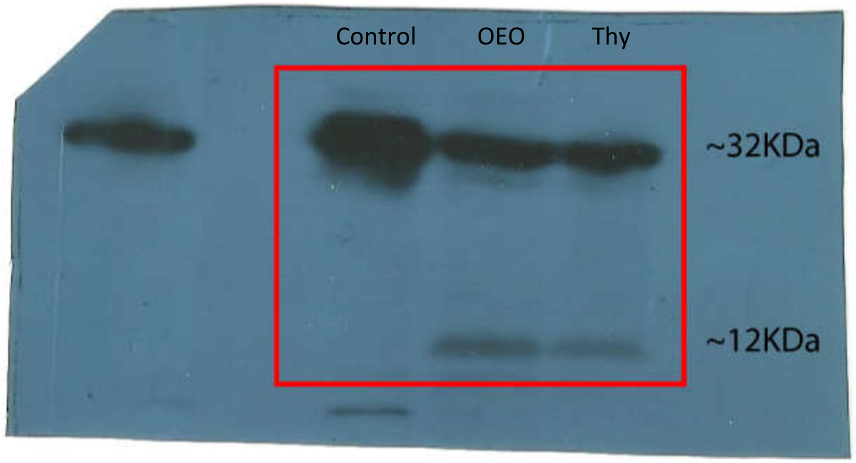

Fig S1: The full length blots or original images for Fig. 9.
